# Observations on Some of the New Hypnotics
*Read at a meeting of the Dorset and West Hants Branch of the British Medical Association.


**Published:** 1887-12

**Authors:** P. Wm. MacDonald

**Affiliations:** Medical Superintendent, Dorset County Asylum


### OBSERVATIONS ON SOME OF THE NEW HYPNOTICS.*

BY

P. Wm. MacDonald, M.D.,

*Medical Superintendent, Dorset County Asylum.*

---

I am induced to read this short paper, believing that the facts hereafter to be mentioned cannot fail to interest you, therapeutically as well as clinically.

In the daily work of your lives as general practitioners, it must often happen that your individual minds are anxiously exercised in the selection of a hypnotic, more especially when you have, as not infrequently happens, to prescribe for the premonitory symptoms of mental disease; and it is hoped that the following short *resumé* of personal and collected facts will assist you in moments of anxiety and great responsibility.

The field of my labours is limited, yet extensive; for although I am denied the compensating advantages of a country doctor's life,—which subject was so ably advocated by our late lamented President (Dr. Dyer),—I am, nevertheless, fully repaid by the opportunities afforded me for studying the physiological effects of any one remedy or remedies over a long series of administrations, which, as you all well know, can only be satisfactorily followed up in an institution.

* Read at a meeting of the Dorset and West Hants Branch of the British Medical Association.

I make use of the expression "some of the recent hypnotics" for I cannot even name, far less write of, many that are from time to time brought under our notice in the pages of the medical journals.

Those I propose shortly discussing are: hyoscyamine, paraldehyde, urethane, and the hydrobromate and hydriodate of hyoscine.

The first, *Hyoscyamine*, is not by any means a recent remedy in the treatment of mental diseases, but is so little used, except by those whose sphere of labours is confined to the wards of an institution for the insane, that I think its efficacy, in the treatment of chronic, as well as recent, cases of mental excitement, should be more generally known; and thus ensure a wider and more varied trial, of a valuable drug, which, in reliable hands, is not more dangerous than several of the older sedatives.

There are two preparations of hyoscyamine in use—the crystal, and a liquid extract. I prefer, and always use the extract. The most reliable preparation of the liquid extract is that known as Mercks's—expensive, but when made into a concentrated solution is easily dispensed, and will retain its strength for a long time. A convenient solution is 1 in 64. The dose of the extract is $\frac{1}{32}$ to $\frac{1}{2}$ gr., but seldom will you have to give more than $\frac{1}{8}$ gr. The disagreeable taste can be overcome by syrup and rose-water, or better still by dropping 4 mins. $=\frac{1}{16}$ gr. of the concentrated solution on a lump of sugar.

In giving hyoscyamine you must select your cases. When wakefulness is the result of surgical or accidental pain its use is not to be recommended, but when due to recurrent cerebral excitement I believe you will find it a useful remedy, and the small dose is at all times a recommendation. Its physiological effects show themselves much quicker than in the case of either bromides or chloral. When given continuously it is apt to accumulate and cause distressing dryness of the mouth and throat and other gastric symptoms.

The cases where you will find its use most serviceable are in strong robust individuals the subjects of acute maniacal excitement, in recurrent attacks of mental fury, and sometimes in the excitement accompanying chronic epilepsy. Give one small dose of $\frac{1}{16}$ gr., to be repeated in two hours if necessary, which is a preferable plan of treatment to the administration of one large dose or keeping the patient continuously under its influence.

Avoid its use in the weak and debilitated.

I must warn you against giving large doses, for although I have not seen an accident from its use, I have been on one or two occasions uncomfortably anxious. Certain well-known authors recommend $\frac{1}{10}$ gr. to be given subcutaneously. I have not practised this plan of administration. Its effects are variable, and you must not expect to have the satisfaction of witnessing rapid and profound sleep in every case.

*Paraldehyde* is comparatively a new hypnotic, and one that promises to be of much interest and value in the treatment of the various causes of wakefulness.

It stands in the same rank as chloral, but is much safer, acts quicker and gives a more natural sleep. Several gentlemen have written in high terms of its utility and safety. Like all new remedies, it is somewhat expensive. The dose is from ʒj to ʒij. The name indicates its oily nature, and this is a difficulty which has to be met in prescribing. It is usually made into a draught by suspending in tragacanth powder, sweetened with syrup. I generally give it with whisky or brandy in the proportion of 1 to 3. The oiliness disappears when mixed with the stimulant, which is I am convinced a most useful adjunct to every hypnotic.

In its action it is gradual, and should it fail to produce sleep, it does not excite. You have a near approach to natural sleep, from which the patient can be easily aroused, and falls off again. Its use is not followed by headache or gastric disturbance, but it continues to be excreted by the lungs for many hours afterwards. This is, by some, considered a great objection. Now it may be and no doubt is an objection, but, as one writer very truly says, "should we not rather look upon the disagreeable breath as a great safeguard against the clandestine use of the drug, and not in the light of an objection?" There is no accumulative principle in paraldehyde, and I have not met with a case of tolerance.

It, however, is a much more valuable hypnotic than many of those now in use; for I believe it can be given with safety and reliance, no matter how abnormal the rhythmical action or other functions of the bodily organs may be.

The class of cases where the use of paraldehyde is indicated is a large one, for if we except the insomnia due to acute mental excitement and severe pain, I believe we may employ it in all other instances of disturbed sleep. I would specially recommend it to your notice in the insomnia of—1, simple mania and melancholia; 2, nervousness and hysteria; 3, profound grief or mental anxiety, and in wakefulness attendant on mental or bodily overwork. One gentleman has prescribed it in gout and rheumatism with inexplicable advantage.

*Urethane* comes to us in crystals—almost tasteless, with a mousy odour, and is easily prescribed. At first the dose was given as 15 grs., but except in cases of heart valvular disease where the patient cannot sleep from "fear," I have not found any dose under 40 grs. to be of use. It is not so certain in its action as paraldehyde, but I believe the after-effects to be less important.

The sleep is natural and refreshing, and in addition to its calmative influence on the nervous centres, it would seem to possess the power of restoring their tonicity, and is therefore to be recommended in the insomnia of aged persons, as well as in cases of heart-lesions.

At present I am somewhat disappointed with urethane. I have tried it in the various phases of mental disease and in many cases of insomnia unconnected with insanity, but cannot now signalise any one class of cases for its special use. Women are more susceptible to its action than men, and in two cases of melancholia occurring at the menopause, I was gratified and pleased with the result of its administration in drachm doses at night.

*Hyoscine.* There are two preparations in use, the hydrobromate and the hydriodate, and as far as my present experience goes they are equally efficacious as a hypnotic. The most convenient solution is 1 in 64 or 1 in 128, and the dose by the mouth ranges from $\frac{1}{128}$ to $\frac{1}{64}$ gr.

I have used it in the various forms of mental disease, but have found it of most service in cases of mania where the loss of sleep was due to continued mental excitement. I have seldom found it to fail, and am of the opinion that hyoscine will at no distant date take a first place among hypnotics.

It is easily prescribed, gives a refreshing sleep, and should not, in proper hands, produce any unpleasant after-effects. It possesses most value as a hypnotic pure and simple. As a general nervine sedative it is not to be compared to the bromides. In insomnia due to melancholia, overwork or fatigue, I have found hyoscine less certain in its action than paraldehyde. In the November No. for 1886 of the *Practitioner* there is an able paper by Dr. Mitchell Bruce on hyoscine, and he there advises us to use it hypodermically. When given hypodermically its action is more rapid than when given by the mouth, but I question whether the latter is not the more preferable plan of treatment in the wards of an Asylum.

In the selection of a hypnotic our first and I may say our dutiful aim should be, to ascertain, often I admit under the greatest difficulty, the true cause of the patient's loss of sleep; and under this head we should note whether the case is one of simple restlessness or genuine wakefulness. In many cases the cause is so apparent as not to need deliberation, but in others it is quite as obscure.

Each of the hypnotics mentioned is equally valuable and reliable, but not in the same class of cases.

Just as we have distinct and easily recognisable remedies, so ought we in prescribing to recognise the widely differing types of insomnia, if we hope and expect to meet with success and reward, and not with disappointment and loss of confidence. Pardon me when I say I am driven to believe that the great and often unhappy difference of opinion—circulating in the ranks of our Profession, as to the value or non-value of a useful remedy, is more frequently due to an error of ours in the selection of cases, than to the inertness of the medicine employed.

When one gentleman of wide experience and high repute tells us that a certain drug has seldom failed him and another equally eminent disapproves of its use, how are we to explain and account for such diversity of opinion, unless we believe that the one who meets with good results gives the proper remedy, and that the other does not—*i.e.*, the one studies his case and prescribes accordingly, but the other does not study his case and prescribes accordingly.

There should be no haphazard practice in the use of hypnotics, nor should we depend too much on well-worn experience, for unless that experience be the result of vigilant, patient and accurate mental application and observation, it is not worthy of the name.

With the question of hypnotics is closely associated the great question of sleep, the physiology and pathology of which we yet know so little. One day the necessary and natural element of health, the next by its absence a symptom of disease.

I feel sure you will agree with me when I say, that seldom is the physician's art more severely taxed than when called upon to restore lost sleep :

> " Sleep that knits up the ravell'd sleave of care,
> The death of each day's life, sore labour's bath,
> Balm of hurt minds, great nature's second course,
> Chief nourisher in life's feast."

